# First fossil record of Discocephalinae (Insecta, Pentatomidae): a new genus from the middle Eocene of Río Pichileufú, Patagonia, Argentina

**DOI:** 10.3897/zookeys.422.6750

**Published:** 2014-06-30

**Authors:** Julián F. Petrulevičius, Yuri A. Popov

**Affiliations:** 1 División Paleozoología Invertebrados, Facultad de Ciencias Naturales y Museo, Universidad Nacional de La Plata, Paseo del Bosque s/n, La Plata (1900), and CONICET, Argentina; 2 Paleontological Institute, Russian Academy of Sciences, Profsoyuznaya str. 123, 117997 Moscow, Russia

**Keywords:** *Acanthocephalonotum* gen. n., Heteroptera, Discocephalini

## Abstract

A new genus and species of Discocephalini, *Acanthocephalonotum martinsnetoi* **gen. n.** et **sp. n.** is described from Río Pichileufú, middle Eocene of Patagonia, Argentina at palaeolatitude ~ 46°S. The new species is the first fossil representative of the Discocephalinae. This taxon is extant in equatorial to subtropical America, and some species reach warm temperate latitudes (Buenos Aires province). The new genus is distinguished from the other genera of Discocephalini by the combination of these characters: interocular width greater than head length; head massive and quadrangular with the anterior margin almost straight; juga touching each other; labrum thick and curved; triangular ante-ocular process extending beyond the eye; broad spine-like antero-lateral process of the pronotum; pronotum explanate and bean shaped; scutellum triangular with a circular tongue reaching the anterior side of abdominal segment 7; and wings well developed with membrane just surpassing end of abdomen.

## Introduction

Pentatomidae is a diverse and globally distributed family of insects with nearly 900 described genera and 4722 living species (Schuh and Slater 1995; Rider 2006, 2011). Their fossil record is less rich, including about 136 species restricted to the Cenozoic (Mitchell 2013) of Asia, Europe, and Central and North America. This distribution is of course due to the presence of palaeoentomologists and intensive collection of fossil insects in these regions. Our fossil belongs to Discocephalinae (Petrulevičius 2008), whose distribution is restricted to the Western hemisphere like three other pentatomoid Neotropical subfamilies: Cyrtocorinae, Edessinae and Stirotarsinae. Until now, the Discocephalinae had no fossil record. The extant forms are restricted to the Neotropical region and represented by 44 genera in Discocephalini and 32 in Ochlerini (Campos and Grazia 2006; Garbelotto et al. 2013; Grazia et al. 2012).

The single specimen comes from the Patagonian locality of Río Pichileufú, Río Negro, Argentina (Petrulevičius 2008). The locality was dated using ^40^Ar/^39^Ar analyses in Wilf et al. (2005) and recalculated in Wilf (2012), giving an age of 47.74 ± 0.05 Ma (million years ago). The locality is renowned for its very high plant diversity (Berry 1938; Wilf et al. 2005, Wilf 2012). Previously reported insects from the same site is the Myrmeciinae ant, *Archimyrmex piatnitzkyi* (Viana and Haedo Rossi 1957; Dlussky and Perfilieva 2003). Other species of *Archimyrmex*, *Archimyrmex smekali* (Rossi de García 1983; Dlussky and Perfilieva 2003) comes from the close but of unknown age locality of Confluencia (Petrulevičius 1999, 2001; Petrulevičius and Martins-Neto 2000). Patagonian Eocene localities are exponentially increasing their known plant and insect diversity in recent years through sustained efforts to collect and describe their fossils (Wilf et al. 2005), giving interesting results in, e.g., Odonata (Frenguelliidae, Austroperilestidae and Aeshnidae; Petrulevičius and Nel 2003, 2005, 2013; Petrulevičius et al. 2010; Petrulevičius 2013) and Mecoptera (Petrulevičius 2005, 2009).

## Material and methods

The fossil is housed at the Museo Asociación Paleontológica Bariloche (repository prefix MAPBAR), San Carlos de Bariloche, Río Negro, Argentina. Recent specimens of Discocephalinae are housed in the Entomological collection (Box 1895) of the Museo de La Plata (MLP), La Plata, Argentina. The holotype of *Glyphuchus sculpturatus* Stål, 1858 is housed in the Naturhistoriska riksmuseet, Stockholm, Sweden.

The fossil and recent specimens from Argentina were photographed with a Nikon D5000 digital camera. The new species was drawn with a camera lucida attached to a Wild M8 stereomicroscope.

## Systematic palaeontology

### Hemiptera Linnaeus, 1758
Heteroptera Latreille, 1810
Pentatomomorpha Leston, Pendergrast & Southwood, 1954
Pentatomoidea Leach, 1815
Pentatomidae Leach, 1815
Discocephalini Fieber, 1860

#### Acanthocephalonotum gen. n.

Taxon classificationAnimaliaHemipteraPentatomidae

http://zoobank.org/C0D67A2B-DDB6-4AC0-86DB-98E23E1D6304

##### Type species.

*Acanthocephalonotum martinsnetoi* sp. n.

##### Diagnosis.

Pronotum with the humeral and posterior angles developed; origin of the labium caudad of the anterior limit of the eyes; head wider than long, anterior margin of head almost straight; labrum thick and curved; juga touching each other before clypeus; interocular width greater than head length (1.16 ×); triangular ante-ocular process extending beyond the eye and perpendicular to the sagittal plane; pronotum with an antero-lateral process (broad spine-like), parallel to the sagittal plane; scutellum triangular with a developed and circular tongue; wings well developed with membrane just surpassing end of abdomen; costal margin bending acutely before end of basal half (boomerang shaped); apex of the scutellum not reaching the apex of corium.

##### Included species.

Type species: *Acanthocephalonotum martinsnetoi* sp. n.

##### Etymology.

From the Latin *acanthus*, meaning spiny, the Greek κεφαλή, meaning head and the Greek νώτος, meaning dorsal and signalling dorsal part of prothorax. “After the head and pronotum with broad spine-like processes”.

#### Acanthocephalonotum martinsnetoi sp. n.

Taxon classificationAnimaliaHemipteraPentatomidae

http://zoobank.org/53C87349-C765-463A-B96D-EA1E5A9896C4

Figs 1 , 2

##### Diagnosis.

Same as for the genus, by monotypy.

##### Description.

The specimen is mainly complete and articulated in dorsal position with a composite view of dorsal and ventral structures.

Body: 4.7 mm long and 3.6 mm wide at pronotum; width (at the base of the hemelytra) / specimen length ratio, 0.78; antennae and legs not visible; head broad, almost rectangular with numerous punctures, wider than long; anterior margin of head almost straight in almost all its width; head 1.15 mm wide in its anterior margin, 0.8 mm long; eyes, 0.24 mm wide, 0.11 mm long; anteocular length 0.36 mm; inter-ocular width 0.95 mm; inter-ocular width / head length ratio, 0.84; distance between ocelli 0.48 mm; distance between eyes and ocellus 0.2 mm; juga (= mandibular plates) touching each other before clypeus; apex of juga contiguous about 0.11 mm; lateral margins of juga deeply concave; clypeus bullet shaped; ante-ocular process extending beyond the eye and perpendicular to the sagittal plane, subtriangular shaped, 0.23 mm long, with its anterior margin convex and posterior margin concave and beside the eye; labrum thick and curved (ventral structure); origin of the labium caudad of the anterior limit of the eyes (ventral structure); pronotum with a broad spine-like antero-lateral process, stout and acute, parallel to the sagittal plane, 0.2 mm long; head length / pronotal width ratio, 0.87; pronotum with numerous punctures, strongly explanate and bean-shaped, 3.6 mm wide, 1 mm long; lateral margins rounded and irregular; scutellum triangular with a developed and circular tongue; scutellum about 2.8 mm wide at base, 1.9 mm long; tongue, 1.2 mm wide and 0.75 mm long; apex of tongue surpassing the corium; apex of scutellum reaching the anterior side of abdominal segment 7; posterior margin of abdominal segment 7 with three straight sides; gonocoxites 8 (ventral structure) with sub-triangular truncate shape, outer lateral margins obliques, posterior ones straights; laterotergites 8 large, sub-triangular, truncate in inner lateral margins.

Wings: well developed membrane just surpassing end of abdomen; corium with punctures; costal margin bending acutely before end of basal half (boomerang shaped); costal angles of corium above ante-penultimate tergum; R slightly curved and followed by punctures by both sides; M slightly zigzagged; CuA almost straight and followed by punctures by both sides; venation not visible in membrane.

**Figure 1.**
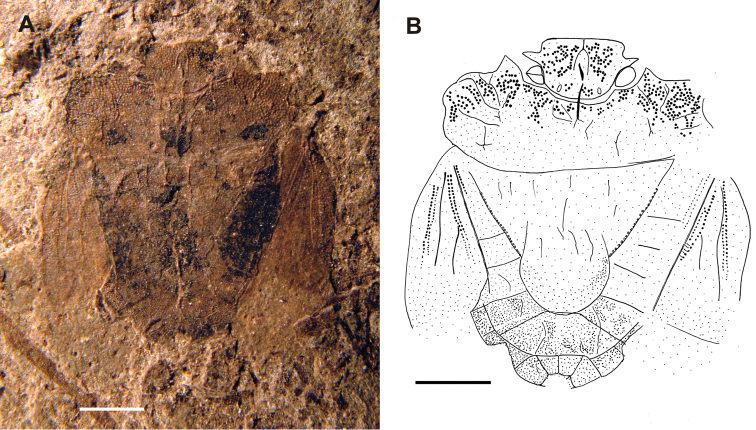
Habitus of *Acanthocephalonotum martinsnetoi* gen. n. et sp. n. Holotype specimen MAPBAR 4137 **A** Photograph **B** line drawing. Scale bars represent 1 mm.

**Figure 2.**
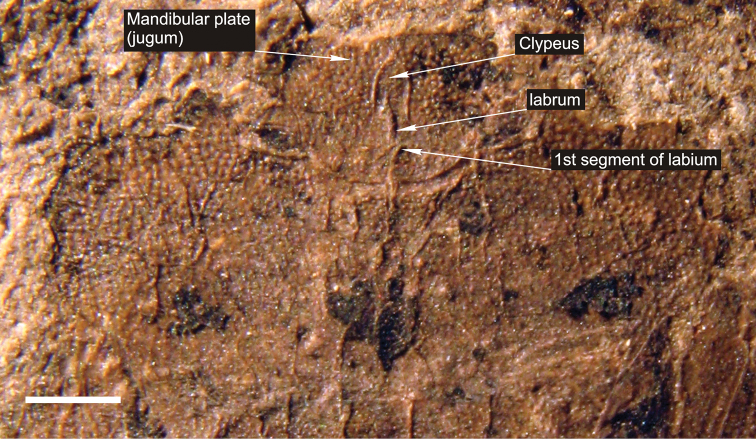
Photograph of detail of head and thorax of *Acanthocephalonotum martinsnetoi* gen. n. et sp. n. Holotype specimen MAPBAR 4137. Scale bar represents 1 mm.

##### Material.

holotype specimen MAPBAR 4137.

##### Type locality and horizon.

Volcanic caldera-lake beds, Río Pichileufú, quarry RP3 (Wilf et al. 2005), province of Río Negro, Patagonia Argentina, palaeolatitude ~ 46°S; middle Eocene (47.7 Ma) (Wilf et al. 2005; Wilf 2012).

##### Etymology.

Dedicated to the memory of Rafael Gioia Martins-Neto, outstanding palaeoentomologist and “irmão de alma”, who unexpectedly and prematurely passed away in 2010 at age 56.

## Discussion

**Figure 3.**
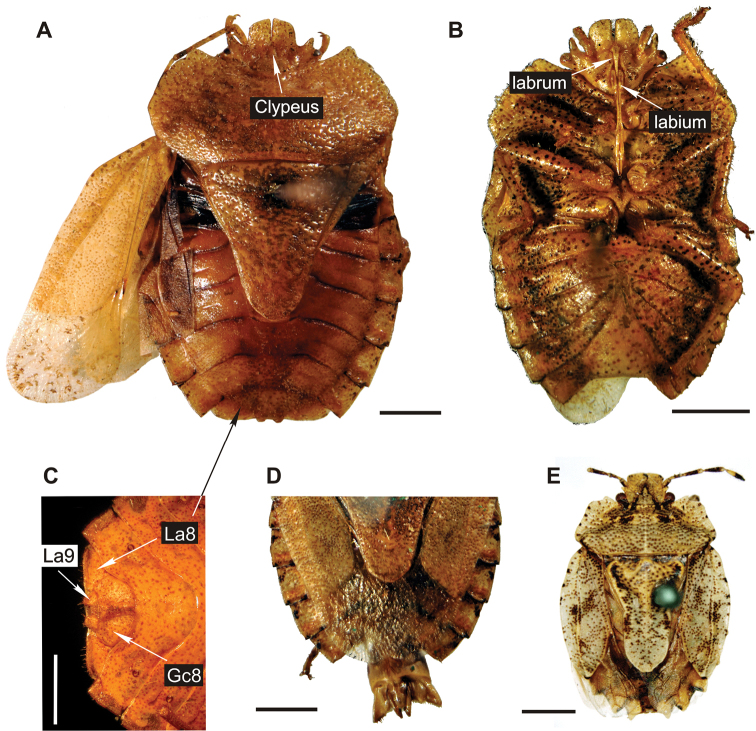
Photographs of habitus of extant genera of Discocephalini **A-D** specimens of *Dryptochephala lurida* Erichson, 1848 **A** female specimen, dorsal view, Tucumán, Argentina **B** male specimen without terminalia, ventral view, Loreto, Misiones, Argentina **C** detail of female abdomen in ventral view, Tucumán, Argentina, Gc8: gonocoxite 8, La8: laterotergite 8, La9: laterotergite 9 **D** detail male abdomen with pygophore in dorsal view, Iguazú, Misiones, Argentina **E** holotype of *Glyphuchus sculpturatus* Stål, 1858, female specimen, dorsal view “Rio Janeiro” (Stål, 1872), Brazil. Scale bars represent 2 mm.

The specimen is a female in dorsal position albeit some ventral structures of head and genitalia are visible resulting in a composite view. Females of Discocephalinae are recognized by having external genital structures as laterotergites and gonocoxites (Fig. 3A, C). Laterotergites 8 are joined by a transverse band visible from the dorsal side (Fig. 3A), but this structure is not visible in the fossil specimen (Figs 1, 2). Males of recent representatives of the group have a pygophore that is easily lost, leaving the free posterior face of the seventh segment with three sides (Fig. 3B, D).

The specimen can be attributed to a species of Pentatomoidea by the presence of several characters (Grazia et al. 2008): pronotum with the humeral and posterior angles developed, scutellum long, general outline of the body ovoid, head dorso-ventrally flattened and laterally carinate, and mandibular plates well developed, reaching or surpassing the clypeus. The first character is considered a synapomorphy of the group by Grazia et al. (2008) (character also present in some Coreidae; Pavel Štys rev. comm.). The new species can be attributed to Pentatomidae: Discocephalinae by having the origin of the labium caudad to the anterior limit of the eyes (Rolston and McDonald 1979; Rolston 1981). It has the inter-ocular width greater (1.2) than the head length. This character is shared with 14 genera of the tribe Discocephalini (Rolston, 1990) called “broadheaded discocephalines”. Rolston (1990) explicitly excluded from this group two genera, *Parvamima* Ruckes, 1960 and *Dryptocephala* Laporte, 1833, because they differ vastly from each other and the other broad-headed genera (Rolston 1990). There are other five broad-headed genera of Discocephalini not discussed by Rolston (1990): *Dentocephala* Ruckes, 1960, *Alcippus* Stål, 1867, *Paralcippus* Becker and Grazia, 1986, *Glyphuchus* Stål, 1860, and *Oncodochilus* Fieber, 1851. Our species is not related to the “broadheaded discocephalines” sensu Rolston (1990) because of its explanate pronotum, quadrangular head and ante-ocular processes. However, the new species shares these characters with *Dryptocephala* (Ruckes, 1966a) and *Glyphuchus* (Stål, 1860). *Parvamima* (Ruckes, 1960) is quite different from our specimen with an almost triangular head and no explanate pronotum. *Dryptocephala* (Figs 3A-D) shares with the described species a strongly explanate bean-shaped pronotum and the ante-ocular processes well developed (Figs 3A, B). Differences between our species and *Dryptocephala* concern the head, which in the fossil is: 1) more massive, 2) quadrangular shape, 3) juga touching each other before the clypeus, 4) anterior margin almost straight in almost all its width, 5) ante-ocular processes perpendicular to the sagittal plane; concerning the pronotum: 6) antero-lateral processes parallel to the sagittal plane; concerning the scutellum: 7) tongue well developed and circular in shape. *Glyphuchus* (Fig. 3d) shares with the described species the massive head, with quadrangular shape, with juga touching each other, and its anterior margin almost straight in almost all its width. Nevertheless, they differ in several characters, i.e., 1) head wider (head length / pronotal width ratio, 0.87 contra 0.79 in *Glyphuchus sculpturatus* Stål, 1858), 2) ante-ocular processes strongly developed, 3) scutellum bean-shaped, and 4) tongue circular shaped. *Glyphuchus* (Fig. 3D) also possesses some special characters, like scutellum explanate but with straight margins (anterior, antero-lateral, lateral, postero-lateral and posterior margins), and laterotergites strongly serrated making a stair-shaped abdomen. Stål (1872) related *Oncodochilus* to *Glyphuchus* because they share a thick and curved labrum which seems to be the case in the described species. *Oncodochilus* also shares with this latter the well developed tongue but with a different shape. *Dentocephala* (Ruckes, 1960) and *Oncodochilus* (Stål, 1860) differ in possessing the ante-ocular processes less developed and lacking an explanate pronotum. Our species could be differentiated from *Oncodochilus*, *Alcippus* (Stål, 1867), and *Paralcippus* (Becker & Grazia, 1986) because it has the head massive, and quadrangular, with the anterior margin almost straight in almost all its width, the ante-ocular processes perpendicular to the sagittal plane, the antero-lateral processes of the pronotum parallel to the sagittal plane, and the explanate pronotum surrounding the head. The last two features are quite similar in *Paralcippus* but the pronotum of that genus is narrower than the abdomen (wider in our specimen) and does not reach the anterior half of the head (reaching the anterior third in our specimen). *Paralcippus* and the new species differ also in the shape of the body (our specimen is much broader) and the tongue (almost quadrangular and incipiently bilobed in *Paralcippus* contra rounded in our specimen).

Other genera that are not broad-headed discocephalines but share other similarities with the new species are *Sympiezorhincus* Spinola, 1837, *Psorus* Bergroth, 1914, *Pelidnocoris* Stål, 1867, *Abascantus* Stål, 1864, and *Coriplatus* White, 1842. They all share a developed tongue (Becker 1977; Becker and Ruckes 1969; Ruckes 1966b; Ruckes and Becker 1970), but the shape is different, being much longer than in the new species. *Psorus* also has an explanate pronotum like the new species but has pedunculate eyes more posteriorly placed and the anterior margin of the head not straight (Fernandes et al. 2008). Considering the differences and unique characters of the new species with respect to the other genera of Discocephalini, we establish a new genus, *Acanthocephalonotum* gen. n.

The Discocephalinae are considered a tropical to subtropical taxon with some species reaching a warm temperate latitudes (Campos and Grazia 2006; Llosa et al. 1990; Grazia et al. submitted; Rider 2011). The southern distribution was Buenos Aires, Argentina (Becker and Grazia 1985; Berg 1884; Grazia et al. submitted) reaching with our discovery the Río Negro province at a palaeolatitude of ~ 46°S.

## Supplementary Material

XML Treatment for Acanthocephalonotum

XML Treatment for Acanthocephalonotum martinsnetoi
